# Covid-19 and spirometry in this age

**DOI:** 10.1186/s13052-022-01199-5

**Published:** 2022-01-18

**Authors:** Angela Klain, Cristiana Indolfi, Giulio Dinardo, Fabio Decimo, Michele Miraglia Del Giudice

**Affiliations:** Department of woman, child and general and specialized surgery, University of Campania ‘Luigi Vanvitelli’, Via Luigi de Crecchio, 4, 80138 Naples, Italy

**Keywords:** Covid-19, Pulmonary function test, Spirometry, Children, Safety

## Abstract

In the last year, many countries adopted a plan to contain hospital infections by Sars-Cov-2 also limiting pulmonary function tests (PFTs), exclusively to indispensable cases. All the recommendations of the major scientific societies regarding the use of PFTs, in particular spirometry, in the Covid era were formulated in the initial period of the pandemic. Currently, the new scientific knowledge about Sars-Cov-2 and the vaccination among healthcare workers, shown new insight to start doing PFTs again to help the investigation and monitoring of patients with respiratory pathology. In this article, we sum up the recommendations of major International Respiratory Societies, and we shared our experience about PFTs in a Pediatric Respiratory Disease Unit during the pandemic.

## Introduction

Respiratory function tests, particularly spirometry, are the main diagnostic tools for most respiratory diseases such as asthma and restrictive lung diseases and are also indispensable for assessing the response to medical therapy and the preoperative risk. With the spread of the new coronavirus (SARS-CoV-2), which causes the COVID-19 pathology, the whole world is facing a health emergency in which, due to the high number of contagions and uncertainties about the infection, many countries have been forced to close PFT laboratories except for urgent cases. Viral diffusion, mainly through aerosols and droplets emitted with the breath [1], is responsible for the great spread of contagions, including nosocomial ones [2].

In children, in most cases, the clinical picture is characterized by a persistent fever, cough, dyspnea, expectoration, myalgias, arthralgias, headache, gastrointestinal symptoms, nasal congestion, and pharyngodynia [3].

There is controversial evidence of the association between allergic diseases and the risk of adverse clinical outcomes of COVID-19 disease. In the survey by Diaferio et al., addressed to Italian pediatricians, for the 75% of responders, a maximum rate of 20% of COVID-19 children were affected by allergic rhino-conjunctivitis, in particular in the North of Italy, while in the Centre and in the South, there was a higher incidence; the 83% of responders affirmed that up to a maximum of 20% of affected children were asthmatic, from 20 to 40% for the 13,5% of responders and from 40 to 60% for the last 3,5%. According to this study, comorbidities as asthma or rhino-conjunctivitis cannot represent a risk factor for more severe disease [4].

Spirometry represents a significant means of contagion of Covid-19, due to the particles emitted with the breath and cough, and, therefore, it is necessary to reassess the role of PFTs tests in Covid era, considering the specific risk/benefit ratio.

Crimi et al., in August 2020, published an article in which they summarized the main international and national guidelines and recommendations regarding the indications for the execution of respiratory function tests in the Covid era and the prevention measures to be adopted by health workers in the PFT laboratories [5]. The main respiratory societies [6–8] agree in stating that PFTs should be performed only in essential cases, postponing the tests in non-imperative cases. The European Respiratory Society (ERS) divides the indications according to the pandemic phase: in phase 1 of the pandemic peak in which there is the maximum prevalence of infections and phase 2 (post-peak), it recommends performing urgent/essential tests only for immediate diagnosis of the disease in progress; while in the post-pandemic phase 3, it is possible to return to pre-Covid reference standards [8]. According to the French Society of Pneumology (SPLF), tests should be performed mainly in the oncological context for the preoperative evaluation and use of pneumotoxic chemotherapy [9]. In Spain, too, Burgos et al. recommend respiratory function tests only in necessary cases, emphasizing the importance of telemedicine in patients with respiratory diseases that can also be managed at home [10]. According to the Portuguese Society of Pneumology (SPP), only tests considered indispensable, such as preoperative and essential for urgent clinical decisions, should be performed [11]. In Australia, the indications are less stringent: The Thoracic Society of Australia and New Zealand (TSANZ) in association with the Australian and New Zealand Society of Respiratory Science (ANZSRS), recommends that all pulmonary function tests should be performed, including cardiopulmonary exercise testing and bronchoprovocation test, in afebrile and asymptomatic for viral disease patients [12]. Even Italian societies dealing with respiratory diseases have expressed their opinion on this issue. The Italian Society of Pneumology (SIP/IRS) recommends a pre-triage by telephone 24–48 h before the appointment, triage immediately before entering the clinic, reception of the patient in respect of social distancing, with control of body temperature and adequate hygiene of the hands; entry into the waiting room only for patients (and their caregiver) equipped with a surgical mask (or equivalent mask with certified filtering activity); rescheduling of appointments ensuring the necessary time spacing for the ventilation of the environments. SIP defines the following indications for respiratory tests: preoperative evaluation for thoracic surgery, pre-transplant evaluation (lung, kidney, liver), COPD first diagnosis, asthma first diagnosis and follow up of severe asthma, interstitial pneumopathies in assessment, prescription of antifibrotic drugs and follow up, follow up of symptomatic “post-COVID” patients or within company protocols or observational studies [13].

The Italian Thoracic Society (ITS) in association with the Italian Society of Hospital Pneumologists (AIPO) in the Position Paper published in May 2020, recommends, in the first phase of the pandemic peak, the performance of functional respiratory tests in necessary situations, especially in preoperative evaluations for thoracic-abdominal surgery, and the monitoring of therapies, whose test results are essential to guide the clinical choice, and the postponement of non-urgent cases, that can be managed at home. In the current phase, post-peak, in which outpatient activities have reopened, they recommend the performance of a careful triage, and only in suspected cases that need a respiratory test, the performance of a nasopharyngeal swab; all other cases should be rescheduled considering the time spacing of appointments [14]. Even the pediatric societies have compiled recommendations on TRFs in the Covid era. According to the Italian Pediatric Respiratory Society (IPRS/SIMRI), respiratory tests, when necessary, should be limited to spirometry and performed only if there are urgent therapeutic decisions in children with chronic pulmonary diseases such as cystic fibrosis, primary ciliary dyskinesia, severe uncontrolled asthma, organ transplantation and onco-hematologic diseases before and after stem cell transplantation [15]. In other cases, the risk/benefit ratio should be evaluated on a case-by-case basis. In suspected or confirmed cases of Covid-19, IPRS indicates postponing testing when at least 2 nasopharyngeal molecular swabs are negative at least 24 h apart.

The world’s leading experts in adult and child respiratory diseases organized a webinar on Virtual International Pediatric Pulmonary Network (VIPPN) [16], as the result of which, in November 2020, they published a paper wherein they analyzed the similarities and differences in indications for PFTs in the World in pediatric age, and the requirements needed to ensure the safety of healthcare personnel and patients in laboratories [17]. According to this article, the indications for performing PFTs in the pediatric age group do not change with the pandemic compared with previous guidelines [18], although each country, in relation to the prevalence of positive cases, may decide to limit testing to essential cases. Contraindications are represented by patients with symptoms/signs of Covid-19 in the past 2 weeks, established or suspected cases of Covid in children in the past month (with at least 7 days of asymptomaticity), or cohabitants in the past 2 weeks.

**Fig. 1:**
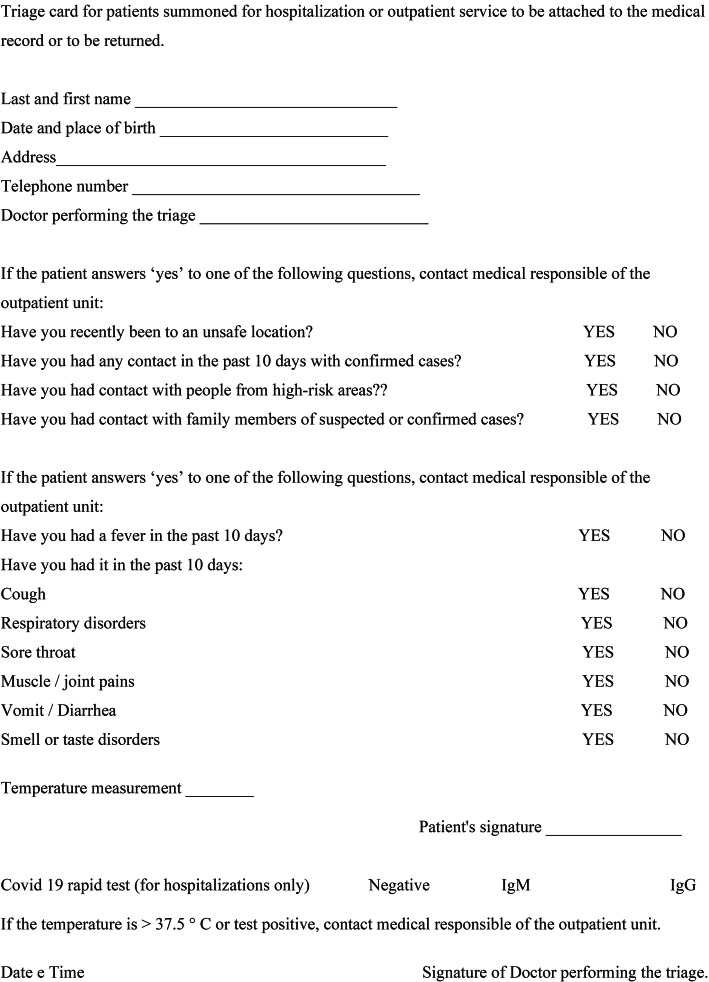
Triage questionnaire (adapted to the English language) delivered to the child and caregiver upon arrival at the clinic

It is important to remark that most of these indications by the international and national respiratory societies of adults and children, date back to more than a year ago and, therefore, they are not updated to the current state in which more precise treatment and prevention protocols have been defined about the pandemic phase and the vaccination campaign of health personnel in Italy has been completed. The experience of the last year has taught us that the containment of SARS-CoV-2 infection is achieved mainly through a valid filter that prevents a not perfectly regulated access to hospitals. Our working organization at the outpatient department of the Pediatric Respiratory Diseases Unit of the University of Campania ‘Luigi Vanvitelli’, during these last months of pandemic, has been based on the limitation of the number of daily visits and a scrupulous triage before entering the clinic, together with the conclusion of the vaccination campaign of health personnel in February 2021. Each visit is scheduled, preventing crowding; children may be accompanied by a single parent. Upon arrival at the clinic, all patients must wear a mask (except for children under 6 years of age) and sanitize their hands. At triage, the child and his or her caregiver are greeted by fully girded health care personnel, who perform different types of procedures depending on the type of service. In the case of an outpatient visit, the body temperature of the child and the caregiver is measured by a no-contact infrared thermometer and questionnaires regarding risk behaviours and contacts with confirmed or suspected cases of Covid and the presence of flu-like symptoms in the last 10 days, are handed out. [Fig. 1].

**Fig. 2:**
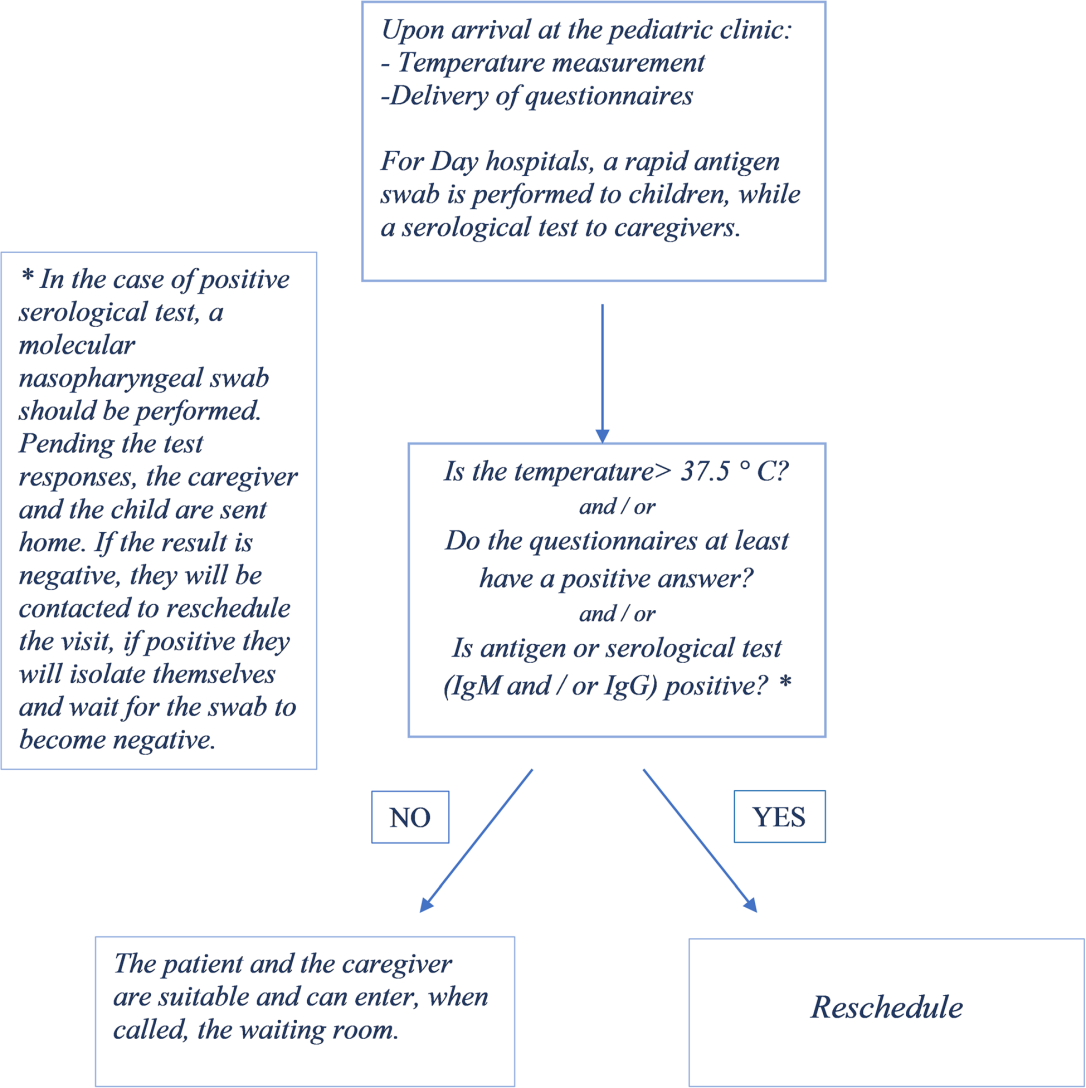
Triage management at the pediatric department of the University of Campania ‘Luigi Vanvitelli’

For all children who must be admitted to the Day Hospital, a nasopharyngeal swab for the rapid antigen test is also performed; while caregivers are subjected to a serological test using finger-stick blood obtained through a lancet. In the case of a positive response to even just one question in the questionnaire, or if the body temperature is higher than 37.5 °C, or a positive antigenic swab, the medical responsible of the outpatient unit is contacted and he will reschedule the outpatient visit or the Day Hospital. In the case of a positive serological test (IgM and/or IgG positive), a nasopharyngeal molecular swab is arranged, and, depending on the outcome, the visit is rescheduled. [Fig. 2].

**Fig. 3:**
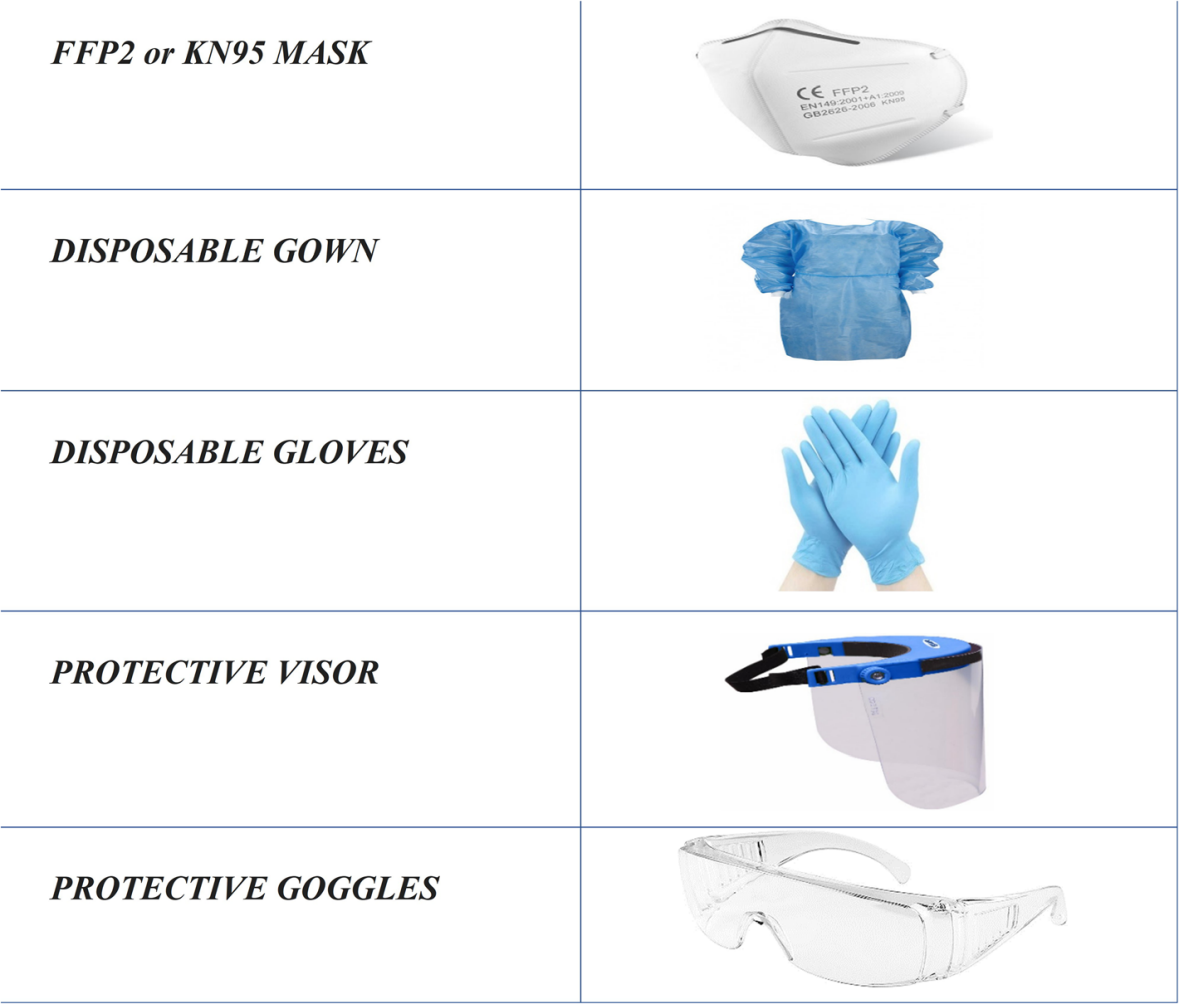
Personal Protective Equipment (PPE) worn by PFT staff

If all questions and the swabs are negative, the child and his / her caregiver are allowed to access our department to a limited extend. Two couples made up of the child and parent enter the waiting room, maintaining an interpersonal distance of 1 m and, when called to enter the clinic, they deliver to the nurses the questionnaires completed and countersigned by the triage staff. All patients with obstructive / restrictive respiratory diseases admitted to Day Hospital or those on an outpatient visit who need it, when called, come in the PFT room to perform spirometry. The spirometry was performed in open space protected by roof. The spirometry was performed by a portable spirometer MIR Spirobank® equipped by MIR’s FlowMir®, a disposable turbine with a paper mouthpiece packaged in individual bags. Each FlowMir® is calibrated and tested before it leaves the factory. The operator was equipped with the following personal protective equipment (PPE): disposable gown and gloves, FFP2 mask, protective visor / goggles. [Fig. 3].

The bronchodilation test was performed using albuterol by metered-dose inhaler (MDI) via the patient’s personal spacer.

## Conclusions

The PFTs are an essential tool for the diagnosis and monitoring of respiratory diseases and the assessment of preoperative risk. The SARS-CoV-2 pandemic raised doubts about the safety of PFTs because of the risk of virus transmission by aerosol droplets and the main scientific societies advised to postpone non-urgent PFTs during the COVID-19 pandemic phase. Currently, the new scientific knowledge about Sars-Cov-2 and the vaccination among healthcare workers, shown new insight to start doing PFTs again to help the investigation and monitoring of patients with respiratory pathology. The use of disposable turbines and the application of safety protocols should be recommended for the correct and safe execution of PFTs in this age.

## Data Availability

Not applicable.
